# Is Lipoprotein(a) Clinically Actionable with Today’s Evidence? The Answer is Yes

**DOI:** 10.1007/s11886-023-01937-z

**Published:** 2023-08-26

**Authors:** Gary S. Ma, Tommy T. Chiou, Michael J. Wilkinson

**Affiliations:** Division of Cardiovascular Medicine, Department of Medicine, Cardiovascular Institute, UC San Diego Health, Sulpizio Cardiovascular Center, University of California San Diego, 9434 Medical Center Dr, MC 7241, La Jolla, CA 92037 San Diego, USA

**Keywords:** Lipoprotein(a), Coronary artery disease, Calcific aortic valve disease, Genetics, Myocardial infarction, Primary prevention

## Abstract

**Purpose of Review:**

Lipoprotein(a) is an independent risk factor for cardiovascular disease. We review the ongoing shifts in consensus guidelines for the testing and management of Lp(a) and provide insight into whether current evidence suggests that awareness and testing of Lp(a) is clinically actionable.

**Recent Findings:**

GWAS and Mendelian randomization studies have established causal links between elevated Lp(a) and forms of CVD, including CAD and calcific aortic valve disease. Testing of Lp(a) identifies patients with similar risk to that of heterozygous FH, enhances risk stratification in patients with borderline/intermediate risk as determined through traditional factors, and facilitates the assessment of inherited CVD risk through cascade screening in patients with known family history of elevated Lp(a). Reductions in Lp(a) through non-targeted therapies including PCSK9 inhibition and lipoprotein apheresis have demonstrated reductions in ASCVD risk that are likely attributable to lowering Lp(a). Targeted therapies to potently lower Lp(a) are in clinical development.

**Summary:**

Lp(a) is actionable, and can be used to identify high risk patients for primary prevention and their family members through cascade screening, and to guide intensification of therapy in primary and secondary prevention of ASCVD.

## Introduction

Lipoprotein(a) [Lp(a)] is a plasma lipoprotein comprised of an apoB100 molecule bound to the glycoprotein apolipoprotein(a) [apo(a)]. Elevated Lp(a) levels have been identified as an independent risk factor for cardiovascular disease (CVD) via mechanisms of increased atherogenesis, thrombosis, and inflammation [1–4]. The emergence of RNA-based therapeutics aimed at potently reducing Lp(a) levels has identified Lp(a) as a key residual risk factor to focus on in the effort to combat lipid-driven atherosclerotic cardiovascular disease (ASCVD) risk. This review aims to provide an examination of the current knowledge of Lp(a) and ongoing shifts in consensus guidelines for the testing and management of Lp(a) and explore the question of whether “Lp(a) is actionable” in the absence of currently available targeted Lp(a)-lowering therapies.

## Lipoprotein(a) and Evidence of Causality in Cardiovascular Disease

Lp(a) is composed of an LDL-like particle, which incorporates an apo(a) molecule via disulfide linkage with the apoB-100 protein on LDL. Apo(a) consists of multiple components, including a protease domain, ten distinct kringle IV units, and one kringle V unit. The length of the apo(a) tail is determined by the number of kringle IV type 2 (KIV2) repeats, which can range from 11 to more than 50 copies [5]. It is noteworthy that the plasma concentration of Lp(a) exhibits an inverse relationship with the size of the apo(a) particle [6]. Elevated Lp(a) is present in about 20% of the population, with higher prevalence seen in African Americans and South Asians as compared with White or East Asian individuals [7–10]. However, it should be noted that the lack of uniformity with which these measurements of Lp(a) were conducted across various studies prevents direct comparison between different ethnic groups. Regardless, Lp(a) plasma levels are estimated to be 70–90% genetically determined through codominant expression of the *LPA* gene on chromosome 6q26-27, leading to the expression of circulating isoforms [11]. Various single nucleotide polymorphisms have been identified to be strongly associated with increased levels of Lp(a) lipoprotein, owing largely to reduced *LPA* copy number and small Lp(a) isoforms [12].

The relationship between elevated Lp(a) and increased CVD risk is well established and understood to be independent of traditional risk factors such as LDL cholesterol (LDL-C) levels. In addition, the putative heritability of Lp(a) serum concentration have led to Mendelian randomization and large genome wide association studies (GWAS) supporting the association between Lp(a) levels and myocardial infarction [12, 13], ischemic stroke [14, 15], peripheral arterial disease [14, 16], and calcific aortic valve stenosis [17–19]. Additionally, large observational epidemiologic studies have established a link between coronary artery disease (CAD) and Lp(a) [1, 20, 21].

## Lp(a) Measurement, Challenges Now and Beyond

Ongoing challenges exist given the lack of uniformity of Lp(a) measurement. Lp(a) serum levels are largely determined using immunoassays with apo(a) specific antibodies. However, given the widely variable size of apo(a) due to differential numbers of KIV2 motif repeats, the accuracy of ELISA-based methods is dependent on the binding sites of the apo(a) specific monoclonal antibodies used, as those specific to KIV2 motif repeats can yield significant variability in the measurement of Lp(a) levels [22]. Furthermore, there are two dominant units for reporting Lp(a) levels, with the first method reporting Lp(a) mass in milligrams per deciliter. ELISA-based methods, calibrated in nanomoles per liter of apo(a), account for the variability in Lp(a) size and therefore report measurements in Lp(a) serum molar concentrations; this method has been recommended by the National Heart, Lung and Blood Institute and likely offers the most accurate quantification method thus far [23].

Furthermore, it is important to note that clinically available laboratory reported LDL-C values (using Friedewald, Martin-Hopkins formula, or direct LDL-C measurement) are limited in that they include Lp(a) cholesterol [Lp(a)-C] within reported LDL-C. Patients with very elevated Lp(a) will carry a greater contribution of Lp(a)-C to LDL-C. While existing methods attempt to estimate LDL-C independent of Lp(a)-C include the Dahlen formula, assuming that Lp(a)-C is a fixed 30% of Lp(a) mass, this was demonstrated to overestimate Lp(a)-C and under-estimate true LDL-C (corrected LDL-C) in patients with elevated Lp(a), and therefore its use is no longer recommended [24]. Alternative methods to directly measure Lp(a)-C and therefore corrected LDL-C have been proposed and offer more accurate estimation of risk reduction attributable to Lp(a) reduction, particularly with forthcoming Lp(a)-specific therapies [25].

The establishment of Lp(a) risk thresholds has been important for facilitating ASCVD risk assessment and therapeutic guidance, with the most common thresholds being 50 mg/dL or ≥ 100–125 nmol/L, since the first threshold was introduced by the European Atherosclerosis Society (EAS) in 2010 [26]. However, more recent recommendations in 2022 now advocate for broader thresholds for ruling in or out Lp(a)-associated CVD risk, with “grey zones” encompassing intermediate values within the range of 30–50 mg/dL or 75–125 nmol/L, likely reflecting the understanding of the continuous, linear risk between Lp(a) and cardiovascular outcomes [27••].

Due to the understanding of significant variations in average Lp(a) values among various racial and ethnic groups, both the National Lipid Association (NLA) and HEART UK associations offered recommendations in their 2019 guidelines acknowledging this racial/ethnic heterogeneity while also noting the limited quality of current evidence supporting the use of population-specific thresholds, and as such recommended a universal cut-point with the caveat that it holds greater clinical relevance in predominantly White populations [28, 29]. In contrast, the American Heart Association (AHA) 2021 statement acknowledged the challenges posed by differences in Lp(a) levels among population ancestries and refrained from providing a specific Lp(a) risk threshold in their guidance [30].

Recent guidelines place emphasis on interpreting Lp(a) elevations in the context of a patient’s global ASCVD risk, with two major studies motivating this shift. The EPIC-Norfolk study stratified 14,051 patients by a composite cardiovascular health metric based on traditional cardiovascular risk factors and found that patients with elevated Lp(a) and metric scores in the healthiest category had only ~ 1/3 of the incident CVD risk compared to those with similar Lp(a) elevations but metric scores in the unhealthiest category [31]. The importance of overall CVD risk in contextualizing Lp(a)-specific risk was redemonstrated in a study of 415,274 individuals of European ancestry in the UK Biobank. In this analysis, patients were stratified by their baseline estimated lifetime CVD risk, and the absolute increase in ASCVD risk associated with elevated Lp(a) was found to be proportional to a patient’s baseline absolute risk [21]. Considering these findings, the EAS in its 2022 statement recommended that the interpretation of Lp(a) levels and subsequent therapeutic decisions be made in the context of a patients’ global ASCVD health after accounting for Lp(a), rather than the Lp(a) levels alone [27••].

## Guidance on Lp(a) Testing from Professional Societies

Prior to 2019, consensus guidelines offered recommendations for testing only in select individuals with elevated risk profiles based on personal or family history of premature ASCVD, heterozygous familial hypercholesterolemia (HeFH), borderline ASCVD risk in the setting of primary prevention, or progressive ASCVD or refractory LDL-C despite optimal therapy. In response to the accumulating evidence linking Lp(a) to CVD, several professional societies have recently issued or updated their clinical guidance regarding Lp(a) with a trend among recent guideline publications towards advocating for universal Lp(a) screening among all adults (2019 European Society of Cardiology/European Atherosclerosis Society (ESC/EAS), 2021 Canadian Cardiovascular Society (CCS) dyslipidemia guidelines, and 2022 EAS Lp(a) consensus statement) [27••, 32, 33] (Table 1).

**Table 1:** Summary of professional society guidelines and statements on Lp(a). Organized by professional society and descending chronologically from most recent by an individual society (EAS Aug 2022) to oldest from an individual society (AHA/ACC Multi-society guideline Nov 2018)

**Guideline**	**Publication date**	
**EAS Lp(a) Consensus Statement**	**Oct 2010**	**Aug 2022**
	*Indications for Lp(a) Testing* Measured once in all adults at intermediate or high risk of CVD/CHD who present with one of: 1) Premature ASCVD 2) FH 3) Family history of premature ASCVD and/or elevated Lp(a) 4) Recurrent CVD despite statin treatment 5) ≥ 3% 10-year risk of fatal ASCVD according to the European guidelines 6) ≥ 10% 10-year risk of fatal and/or non-fatal CHD according to the US guidelines	*Indications for Lp(a) Testing* 1) All adults, at least once. Repeats not necessary except in setting of liver or kidney disease, or acute infection 2) Cascade testing in the setting of FH, family history of very high Lp(a), or personal or family history of ASCVD
	*Lp(a) Risk Thresholds* 1) The association between Lp(a) and ASCVD is continuous without a threshold or dependence on LDL-C or non-HDL-C levels 2) Desirable levels for Lp(a) < 50 mg/dL (~ 80^th^ percentile in White individuals)	*Lp(a) Risk Thresholds* 1) Continuous relationship between Lp(a) and ASCVD risk 2) Recommend using Lp(a) thresholds with ‘grey’ zones (e.g., 30–50 mg/dL or 75–125 nmol/L) to either rule-in (≥ 50 mg/dL; 125 nmol/L) or rule-out (< 30 mg/dL; 75 nmol/L) Lp(a)-related ASCVD risk 3) Very high Lp(a) levels (> 180 mg/dL or > 430 nmol/L) identify individuals with lifetime ASCVD risk equivalent to untreated HeFH
	*Management of Elevated Lp(a)* Reduction in Lp(a) should mainly be achieved using niacin (1-3 g/day)	*Management of Elevated Lp(a)* 1) Recommend early, comprehensive management of all ASCVD risk factors (including LDL-C, blood pressure, glucose, and lifestyle factors) as guided by patient’s absolute global ASCVD risk and Lp(a) level 2) Consider lipoprotein apheresis in patients with very high Lp(a) and progressive cardiovascular disease despite optimal management of risk factors 3) Niacin is not recommended for Lp(a) lowering
**AHA Scientific Statement**	**Oct 2021**	
	*Indications for Lp(a) Testing* 1) May be appropriate for additional risk stratification in patients with borderline (5%–7.4%) or intermediate (7.5%–19.9%) 10-year ASCVD risk as estimated by well-validated equation for the patient population 2) Cascade screening of family members of patients with elevated Lp(a) may identify additional individuals with elevated Lp(a) because of its autosomal codominant inheritance pattern	
	*Lp(a) Risk Thresholds* 1) No threshold specified. Cited heterogeneity in Lp(a) assay specifications, Lp(a) differences across different population ancestries, and the importance of patient comorbidities in interpreting significance of Lp(a) levels as reasons universal threshold could not be established 2) Recommend incorporating Lp(a) into ASCVD risk estimation	
	*Management of Elevated Lp(a)* 1) To be interpreted as a risk-enhancing factor that further informs the 10-year ASCVD risk estimate (predicted 10-y risk × [1.11^(patient’s Lp(a) level in nmol/L/50)^] 2) Management based on updated 10-year ASCVD risk estimate per ACC/AHA’s 2019 guideline on primary ASCVD prevention	
**NLA Scientific Statement on Lp(a)**	**Apr 2019** *	**Sep 2021**
	*Indications for Lp(a) Testing* Reasonable in individuals with: 1) Family history of first-degree relatives with premature ASCVD (< 55 y of age in men; < 65 y of age in women) 2) Premature ASCVD, especially in absence of traditional risk factors 3) Primary severe hypercholesterolemia (LDL-C ≥ 190 mg/dL) or suspected FH 4) Very-high-risk for ASCVD (i.e. those with a history of multiple major ASCVD events or 1 major ASCVD event and multiple high-risk conditions) to better define those who are more likely to benefit from PCSK9 inhibitor therapy May be reasonable in individuals with: 1) Intermediate (7.5%-19.9%) 10-y ASCVD risk when the decision to use a statin is uncertain, to improve risk stratification in primary prevention 2) Borderline (5%-7.4%) 10-y ASCVD risk when the decision to use a statin is uncertain, to improve risk stratification in primary prevention 3) Less-than-anticipated LDL-C lowering, despite good adherence to LDL-C lowering therapy 4) Family history of elevated Lp(a) 5) Calcific valvular aortic stenosis 6) Recurrent or progressive ASCVD, despite optimal lipid-lowering therapy	*Indications for Lp(a) Testing* Referenced the NLA 2019 indications [28] and additionally noted that Lp(a) measurement may be valuable for guiding management in: 1) Patients with a strong family history of ASCVD 2) Patients who do not fully respond to statin therapy 3) Patients who go on to have an ASCVD event while on evidence-based lipid-lowering therapy 4) Patients who are already on maximal dose statin therapy ± ezetimibe, whose LDL-C remains above 70 mg/dL, to determine those who may benefit from PCSK9 inhibitor therapy
	*Lp(a) Risk Thresholds* Reasonable to use Lp(a) ≥ 50 mg/dL or ≥ 100 nmol/L as levels suggesting increased risk in White patients	*Lp(a) Risk Thresholds* Not discussed
	*Management of Elevated Lp(a)* 1) Among adults aged 40–75 y with a 10-y ASCVD risk of 7.5%-19.9%, use Lp(a) ≥ 50 mg/dL or ≥ 100 nmol/L as risk-enhancing factor to favor initiation of a moderate- or high-intensity statin in those with on-treatment LDL-C ≥ 70 mg/dL (or non–HDL-C ≥ 100 mg/dL) 2) Among adults at high risk (i.e., those with clinical ASCVD) or at very-high risk (i.e., adults with a history of multiple major ASCVD events or 1 major ASCVD event and multiple high-risk conditions), consider more intensive LDL-C lowering to achieve greater ASCVD risk reduction 3) Among very-high-risk patients taking a maximally tolerated statin, with Lp(a) ≥ 50 mg/dL or ≥ 100 nmol/L and LDL-C ≥ 70 mg/dL (or non–HDL-C ≥ 100 mg/dL), add ezetimibe. This approach may also be reasonable among high-risk patients 4) Among very-high-risk patients taking a maximally tolerated statin and ezetimibe, with an Lp(a) of ≥ 50 mg/dL or ≥ 100 nmol/L and LDL-C ≥ 70 mg/dL (or non–HDL-C ≥ 100 mg/dL), add a PCSK9 inhibitor 5) Niacin is not recommended to reduce ASCVD risk in patients taking moderate- to high-intensity statins ± ezetimibe with an on-treatment LDL-C < 80 mg/dL	*Management of Elevated Lp(a)* Not discussed
**CCS Dyslipidemia Guideline**	**Jul 2016**	**Aug 2021**
	*Indications for Lp(a) Testing* Lp(a) might aid risk assessment in subjects with intermediate Framingham Risk Score or with a family history of premature coronary artery disease	*Indications for Lp(a) Testing* 1) All persons at initial lipid screening 2) Especially important in younger patients with a very strong family history of premature ASCVD
	*Lp(a) Risk Thresholds* ASCVD risk is increased by approximately twofold in patients with Lp(a) > 30 mg/dL	*Lp(a) Risk Thresholds* 1) The risk of ASCVD increases with increasing Lp(a) levels > 30 mg/dL in a dose-dependent fashion 2) Threshold of Lp(a) ≥ 50 mg/dL (or ≥ 100 nmol/L) for primary prevention
	*Management of Elevated Lp(a)* Not discussed	*Management of Elevated Lp(a)* Primary prevention (Lp(a) ≥ 50 mg/dL (or ≥ 100 nmol/L)): 1) For all patients, recommend more intensive health behavior modification counseling and management of other ASCVD risk factors 2) For intermediate-risk patients and/or low-risk patients with LDL-C between 3.5–5 mmol/L (~ 135–194 mg/dL), recommend further ASCVD risk assessment (including age-appropriate vascular imaging for detection of subclinical atherosclerosis) and earlier introduction of statins or other lipid-lowering therapy Secondary prevention: Recommend intensification of lipid-lowering therapy with PCSK9 inhibitors for patients with Lp(a) level ≥ 60 mg/dL (120 nmol/L)
**AACE/ACE Dyslipidemia Consensus Statement**	**Oct 2020**	
	*Indications for Lp(a) Testing* 1) All patients with clinical ASCVD, especially premature or recurrent ASCVD despite LDL-C lowering 2) Individuals with a family history of premature ASCVD and/or increased Lp(a) 3) Individuals with South Asian or African ancestry, especially those with a family history of premature ASCVD and/or increased Lp(a) 4) Individuals with a 10-year ASCVD risk ≥ 10% (primary prevention setting), in order to stratify risk 5) Patients with a personal or family history of aortic valve stenosis 6) Patients with refractory elevations of LDL-C despite aggressive LDL-C-lowering therapy (i.e., statin resistance)	
	*Lp(a) Risk Thresholds* A Lp(a) level > 50 mg/dL is associated with increased risk of recurrent events in patients on statin therapy	
	*Management of Elevated Lp(a)* Aggressive LDL-C lowering.	
**HEART UK Consensus Statement**	**Oct 2019**	
	*Indications for Lp(a) Testing* Measured once among those with: 1) Personal or family history of premature ASCVD (< 60 years) 2) First degree relatives with raised serum Lp(a) levels (> 200 nmol/L) 3) FH, or other genetic dyslipidemias 4) Calcific aortic valve stenosis 5) Borderline increased (but < 15%) 10-year risk of a cardiovascular event	
	*Lp(a) Risk Thresholds* ASCVD risk conferred by Lp(a): Minor: 32–90 nmol/L Moderate: 90–200 nmol/L High: 200–400 nmol/L Very high: > 400 nmol/L	
	*Management of Elevated Lp(a)* Recommendations for those with Lp(a) > 90 nmol/L: 1) Reduce overall ASCVD risk 2) Control hyperlipidemia (goal non-HDL-C < 100 mg/dL (2.5 mmol/L)) 3) Consider lipoprotein apheresis as per the 2008 HEART UK Lipoprotein apheresis statement (i.e., patients with progressive coronary disease and Lp(a) greater than ~ 150 nmol/L (> 60 mg/dL) whose LDL-C remains > 125 mg/dL (3.3 mmol/L) despite maximal lipid-lowering therapy)	
**ACC/AHA Primary Prevention Guideline**	**Sep 2019**	
	*Indications for Lp(a) Testing* Relative indication: family history of premature ASCVD (males, age < 55 y; females, age < 65 y)	
	*Lp(a) Risk Thresholds* Lp(a) ≥ 50 mg/dL or ≥ 125 nmol/L constitutes a risk enhancing factor, especially at higher levels of Lp(a)	
	*Management of Elevated Lp(a)* Same as the 2018 AHA/ACC Multi-society Cholesterol Guideline	
**ESC/EAS Dyslipidemia Guideline**	**Aug 2016**	**Aug 2019**
	*Indications for Lp(a) Testing* Not recommended for risk screening in the general population. Consider in individuals with: 1) Premature ASCVD 2) FH 3) Family history of premature ASCVD or elevated Lp(a) 4) Recurrent ASCVD despite optimal lipid-lowering therapy 5) > 5% 10-year risk of fatal ASCVD based on SCORE	*Indications for Lp(a) Testing* All adults, at least once
	*Lp(a) Risk Thresholds* Risk is regarded as significant when Lp(a) is above the 80^th^ percentile (50 mg/dL)	*Lp(a) Risk Thresholds* 1) Patients with Lp(a) > 180 mg/dL (> 430 nmol/L) may have similar lifetime ASCVD risk as those with HeFH 2) Patients with less extreme Lp(a) elevations may still be at higher risk for ASCVD that is not reflected by other lipid or lipoprotein measurements (no specific Lp(a) threshold mentioned)
	*Management of Elevated Lp(a)* 1) Intensified treatment of the modifiable risk factors, including LDL-C 2) Consider PCSK9i antibody if patient has high Lp(a) and FH	*Management of Elevated Lp(a)* Not discussed
**AHA/ACC Multi-society Cholesterol Guideline**	**Nov 2018**	
	*Indications for Lp(a) Testing* Relative indications: 1) Family history of premature ASCVD (males, age < 55 y; females, age < 65 y) 2) Personal history of ASCVD not explained by major risk factors	
	*Lp(a) Risk Thresholds* An Lp(a) ≥ 50 mg/dL or ≥ 125 nmol/L constitutes a risk-enhancing factor especially at higher levels of Lp(a)	
	*Management of Elevated Lp(a)* If Lp(a) ≥ 50 mg/dL or ≥ 125 nmol/L, utilize as a risk enhancing factor in the context of absolute global ASCVD risk assessment	

*AACE* American Association of Clinical Endocrinologists, *ACC* American College of Cardiology, *ACE* American College of Endocrinology, *AHA* American Heart Association, *ASCVD* atherosclerotic cardiovascular disease, *CCS* Canadian Cardiovascular Society, *CHD* coronary heart disease, *CVD* cardiovascular disease, *EAS* European Atherosclerosis Society, *ESC* European Society of Cardiology, *FH* familial hypercholesterolemia, *HeFH* heterozygous familial hypercholesterolemia, *LDL-C* low-density lipoprotein cholesterol, *NLA* National Lipid Association

*Reprinted in Sep 2022 without change in content

Proponents of universal screening cite the high epidemiologic burden of elevated Lp(a) and increasing evidence linking Lp(a) to ASCVD, as well as the ability to enhance ASCVD risk assessment. In contrast, the 2019 NLA scientific statement recommended against testing in the general population due to the lack of currently available targeted Lp(a)-lowering therapies and insufficient evidence linking Lp(a)-specific treatments to improved outcomes [28]. Nevertheless, some societal guidelines highlight that universal screening carries likely little harm and identification of patients with extremely high Lp(a) levels ≥ 180 mg/dL (≥ 430 nmoL/L) is important as these individuals have lifetime ASCVD risk similar to patients with HeFH [33]. Recommendations may continue to shift toward universal screening for Lp(a) in the general population should targeted Lp(a) lowering therapies demonstrate benefit in lowering CVD risk.

Furthermore, patients in the highest tertiles of Lp(a) and oxidized phospholipids on apoB (OxPL-apoB) exhibit more rapid hemodynamic deterioration and need for aortic valve replacement [34–36]. Several societies have proposed that Lp(a) examination may inform the frequency of valve surveillance in those patients with established calcific aortic valve disease (CAVD) [28, 29, 37].

## Mitigation of Lp(a)-Driven CVD Risk

There are no currently approved Lp(a) lowering therapeutics available for clinical use, and thus the consensus recommendation among professional societies for Lp(a) management centers primarily on tighter control of other traditional ASCVD risk factors, including lowering LDL-C, blood pressure, blood glucose, and promotion of improved dietary/lifestyle changes. Intensification of such changes, irrespective of their effect on Lp(a) levels, can mitigate some ASCVD risk as those with elevated Lp(a) in ideal cardiovascular health had lower risk than those not following healthy lifestyle [31].

As new data have emerged on how Lp(a) and other risk factors impact overall ASCVD risk, recent guidelines have started providing more detailed recommendations. The 2022 EAS guideline, for instance, has outlined targets for LDL-C reduction required to mitigate Lp(a)-associated risk based on different Lp(a) levels and the age at which LDL-C-lowering therapy is initiated [27••]. While these recommendations suggest the potential for some patients to mitigate their Lp(a)-related risk through aggressive management of other factors, this approach has important limitations and may be inadequate for individuals whose primary ASCVD risk stems disproportionately from elevated Lp(a). For example, work by Trinder et al. [38] suggests that apoB is insufficient to explain Lp(a)-driven risk for CAD, and therefore it is unclear whether aggressive LDL-C and/or apoB lowering can fully offset risk from Lp(a). Importantly, statin therapy may lead to increases in Lp(a) [6, 39]. In a large meta-analysis of seven, randomized placebo-controlled statin outcomes trials, ASCVD risk persisted in a linear relationship with Lp(a) despite statin treatment [40]. However, despite the potential for statins to increase Lp(a) levels, statin therapy remains a cornerstone of ASCVD prevention and risk reduction and statins remain first-line lipid-lowering therapy for ASCVD risk reduction including for patients with elevated Lp(a).

Beyond the general recommendation for traditional risk factor control, the use of PCSK9 inhibitor (PCSK9i) monoclonal antibodies (mAbs) for managing elevated Lp(a) has been shown to reduce Lp(a) levels by up to 15–30% [41, 42]. Post hoc analyses of the FOURIER and ODYSSEY OUTCOMES clinical trials showed that patients with elevated Lp(a) (defined as Lp(a) > 50 mg/dL in the post-hoc FOURIER study, Lp(a) > 60 mg/dL in the post-hoc ODYSSEY OUTCOMES study) derived greater absolute risk reduction from PCSK9i mAb treatment compared to those with Lp(a) levels below the threshold. Further analysis of the ODYSSEY OUTOMES study suggested that the Lp(a)-lowering effect of alirocumab contributed independently to ASCVD risk reduction [43•]. In keeping with these findings, the NLA dyslipidemia guideline suggested consideration of PCSK9 mAb for very high-risk patients taking a maximally tolerated statin and ezetimibe, with an Lp(a) of ≥ 50 mg/dL (or ≥ 100 nmol/L) and LDL-C ≥ 70 mg/dL (or non–HDL-C ≥ 100 mg/dL) [28]. Similarly, the CCS in 2021 recommended consideration of PCSK9i mAb for secondary prevention in high-risk patients with Lp(a) > 60 mg/dL [32].

Lipoprotein apheresis has also been examined as a therapeutic option in a number of single arm studies, with reductions in Lp(a) levels by up to 75% (time averaged reduction of 30–35%), with some studies suggesting associated reductions in CVD events [44–46]. However, apheresis remains an invasive and expensive procedure, thereby limiting its widespread use. Nevertheless, guidelines have suggested utility for this modality in select patients with progressive coronary artery disease and Lp(a) greater than 150 nmol/L (> 60 mg/dL) and whose LDL-C remains greater than 125 mg/dL despite maximally tolerated lipid lowering therapy [27••, 29]. In the USA and Germany, a current indication for lipoprotein apheresis is HeFH with LDL-C ≥ 100 mg/dL and Lp(a) ≥ 60 mg/dL and either CAD or peripheral artery disease (PAD) [47, 48].

Niacin has been shown to reduce Lp(a) levels by up to 30%, though concerns over tolerability as well as the unknown cardiovascular benefit have limited recommendations for its use [49, 50].

Finally, antisense oligonucleotides (pelacarsen) targeting apo(a) and investigational small interfering RNA molecules targeting apo(a) RNA are in clinical trials (e.g., olpasiran and SLN360) [51–53]. These investigational, targeted therapies have achieved potent serum Lp(a) lowering by up to 80–100% at the highest tested doses, in contrast with more modest Lp(a) reductions demonstrated with currently available therapies, such as PCSK9i.

## Is Lp(a) Clinically Actionable?

Our perspective is that Lp(a) has important clinical implications that are actionable with the current literature on multiple levels. Despite the lack of targeted therapies for Lp(a)-driven CVD risk reduction in clinical practice, the expansion of the knowledge base surrounding Lp(a) and its conferred risks for various CVD profiles in recent years offers new insights into how more widespread testing of Lp(a) is of greater utility than previously realized. Shifting consensus among various societal guidelines has also reflected the growing body of evidence that understanding Lp(a)-driven risk in the broader sense is of utility in a number of clinical scenarios [27••, 32, 33].

### Primary Prevention of ASCVD

The utility of Lp(a) screening in primary prevention is multifold, and our perspective is that universal testing should be considered in all adults at least once, as suggested in several recent statements from professional societies [27••, 32, 33]. Lp(a) testing provides a practical method in identifying these high CVD risk patients for aggressive lipid lowering therapy, particularly as evaluation of traditional risk factors may not capture these patients. Furthermore, certain racial/ethnic backgrounds demonstrate higher prevalence for elevated Lp(a), including those of African or South Asian heritage, and the identification of elevated Lp(a) in patients of South Asian or Latin descent may support maximizing lifestyle modifications to reduce CVD if demonstrated to have Lp(a) > 50 mg/dL, particularly as these populations may have the highest Lp(a) attributable risk for myocardial infarction (MI), independent of other traditional risk factors [54]. Finally, both the 2018 Multi-Society Cholesterol Guideline and the 2019 American College of Cardiology (ACC)/American Heart Association (AHA) Primary Prevention Guideline recommend the use of Lp(a) as a CVD “risk enhancer” among those with borderline and intermediate risk as determined by the 10-year Pooled Cohort Equation. Together with additional evidence-based tools for risk stratification (e.g., coronary artery calcium scoring), elevated Lp(a) may inform discussions with patients on diet/lifestyle counseling and consideration of the initiation and/or intensification of statin therapy and non-statin lipid-lowering therapy [55, 56]. In primary prevention, the use of aspirin has also been associated with potential benefit in patients with elevated Lp(a)-associated genotypes as demonstrated in secondary analyses of the ASPREE trial and Women’s Health Study [57, 58]. Further investigation with randomized trials will help define the role of aspirin in primary prevention of ASCVD in patients with elevated Lp(a).

### Secondary Prevention of ASCVD

While post hoc analyses of FOURIER and ODYSSEY OUTCOMES suggest a potential benefit of Lp(a) lowering in the secondary prevention of ASCVD, data from randomized, controlled cardiovascular outcomes trials with potent Lp(a)-lowering therapies (HORIZON NCT04023552 and OCEAN(a) NCT05581303) are poised to better examine the ability of Lp(a)-lowering to reduce ASCVD risk. Until such data are available, however, the examination of Lp(a) levels may be particularly beneficial in two populations: 1) patients with recurrent ASCVD events despite aggressive lipid lowering therapy and 2) patients with less than expected LDL-C lowering on evidence-based lipid lowering therapy [28, 37]. Identification of patients with elevated Lp(a) in this context may facilitate practicing clinicians to pursue aggressive CVD risk factor management, consideration of the lowest guideline recommended LDL-C targets, and consideration of the use of PCSK9 inhibitors or lipoprotein apheresis in select cases. Finally, in patients with established CAVD, Lp(a) may help identify patients who may stand to benefit from more aggressive aortic valve surveillance, given the association of more rapid valve deterioration and need for replacement in those at the highest tertiles of Lp(a) levels [29, 37].

**Fig. 1:**
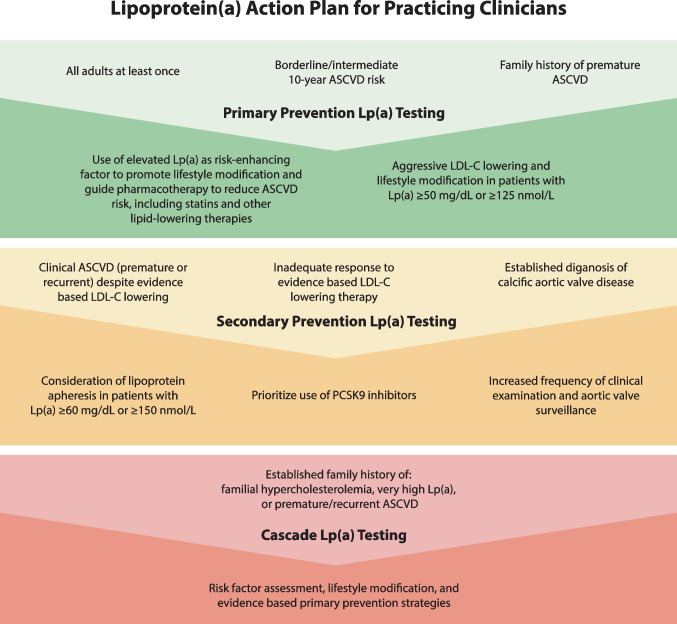
Proposed lipoprotein(a) action plan for practicing clinicians

Furthermore, given that Lp(a) levels are largely determined by genetic factors, with little influence from lifestyle modifications, cascade testing in patients with either premature ASCVD and/or known elevated Lp(a) may help prognosticate the CVD risk profiles of offspring and other family members and help direct lifetime risk reduction and primary prevention [27••, 30]. These recommendations are summarized in the proposed “Lp(a) action plan” for clinicians (Fig. 1).

## Conclusions

In summary, despite the current lack of targeted therapies for Lp(a) lowering, Lp(a) is clearly actionable today. Recommendations for more widespread Lp(a) testing continue to gain traction with the goal of helping to refine our ability to estimate CVD risk in patients and families, and guide treatment decisions in primary and secondary prevention of ASCVD.
